# Case Report: Two Is Not (Always) Better Than One: Pyloric Gland Adenoma of the Gastric Cardia and Concurrent Neuroendocrine Cell Dysplasia Arising From Autoimmune Gastritis

**DOI:** 10.3389/fmed.2022.890794

**Published:** 2022-05-19

**Authors:** Camilla Guerini, Marco Vincenzo Lenti, Chiara Rossi, Giovanni Arpa, Andrea Peri, Anna Gallotti, Antonio Di Sabatino, Alessandro Vanoli

**Affiliations:** ^1^Unit of Anatomic Pathology, Department of Molecular Medicine, University of Pavia, and IRCCS San Matteo Hospital Foundation, Pavia, Italy; ^2^First Department of Internal Medicine, IRCCS San Matteo Hospital Foundation, University of Pavia, Pavia, Italy; ^3^Unit of Pathology, Istituti Clinici Scientifici Maugeri IRCCS, Pavia, Italy; ^4^Department of Surgery, IRCCS San Matteo Hospital Foundation, University of Pavia, Pavia, Italy; ^5^Unit of Radiology, Department of Intensive Medicine, IRCCS San Matteo Hospital Foundation, University of Pavia, Pavia, Italy

**Keywords:** autoimmune gastritis, pyloric gland adenoma, ECL cell dysplasia, gastric carcinogenesis, immune microenvironment

## Abstract

Autoimmune gastritis is a chronic immune-mediated disorder characterized by varied clinical manifestations and that should be endoscopically managed over time, as the gastric atrophy contributes to microenvironmental alterations of the stomach milieu, and an increased cancer risk has been linked to this condition. Here, we report the unusual case of a woman who developed a cardiac high-grade pyloric adenoma in a context of previously undiagnosed autoimmune gastritis with synchronous neuroendocrine cell hyperplastic and dysplastic lesions.

## Introduction

Autoimmune gastritis (AIG) is an immune-mediated, chronic, organ-specific disorder characterized by the destruction of oxyntic mucosa, mediated by anti-parietal cell antibodies (PCA) which recognize the subunits of the H+/K+ ATPase, a proton pump that can be found in the parietal cells (1). The net result is oxyntic mucosal inflammation and its progression to corpus-fundus mucosal atrophy. The progressive destruction of hydrochloric acid- and intrinsic factor-producing parietal cells also elicits a simultaneous hyperplasia of the gastrin-producing cells of the antrum and, subsequently, of the neuroendocrine enterochromaffin-like (ECL) cells of the oxyntic mucosa. The histological progression from simple, linear, micronodular and adenomatous hyperplasia, to dysplasia and, eventually, to type 1 gastric neuroendocrine tumor (NET) has been extensively described (2–4).

The neuroendocrine lineage is not the only one interested by the pro-carcinogenic processes in the context of AIG. In fact, the glandular atrophy that characterizes this autoimmune process is accompanied by metaplastic changes of the oxyntic mucosa with its progressive “antralization” and the development of pseudo-pyloric and intestinal metaplasia. These atrophic and metaplastic changes are associated with an increased risk of development of pre-invasive and invasive neoplasms of epithelial-glandular origin, even if the cell of origin has not been recognized with certainty (5). Other factors may also play a role in favoring the progression of atrophy into epithelial dysplasia and cancer, including genetic predisposition, a specific proteomic signature, and gastric microbiota alterations (6, 7).

Pyloric gland adenomas (PGA) are rare mass-forming neoplasms composed of closely packed pyloric glands, lined by columnar or cuboidal cells with pale or eosinophilic cytoplasm and round nuclei without prominent nucleoli, showing a characteristic immunoreactivity for MUC5AC and MUC6, respectively in the superficial and deep glandular components (8–11). Although they can be diagnosed in several organs, almost always in association with gastric heterotopia or foveolar- and pyloric-type metaplasia (12), the predominant site of presentation is the gastric corpus in patients with an underlying AIG. These lesions are characterized by a 50% risk of concomitant gastric adenocarcinoma, even though submucosal invasion occurs in only 10% of the cases (11).

In this paper we report the case of a 79-year-old woman who underwent a partial gastric resection for a pyloric gland adenoma which arose in an unusual site (cardia) on a background of undiagnosed AIG and was associated with neuroendocrine cell hyperplasia and dysplasia in the adjacent fundic mucosa.

### Case Presentation

The patient has been suffering from essential hypertension since roughly the age of 50, and she has been on an angiotensin converting enzyme inhibitor since then. Also, she has been suffering from autoimmune thyroid disease, namely Hashimoto's thyroiditis. The patient has a first-degree family history of various cancer types, including breast cancer (mother), endometrial cancer (daughter), and lung cancer (father). She has never smoked, nor is she a drinker.

**Figure 1:**
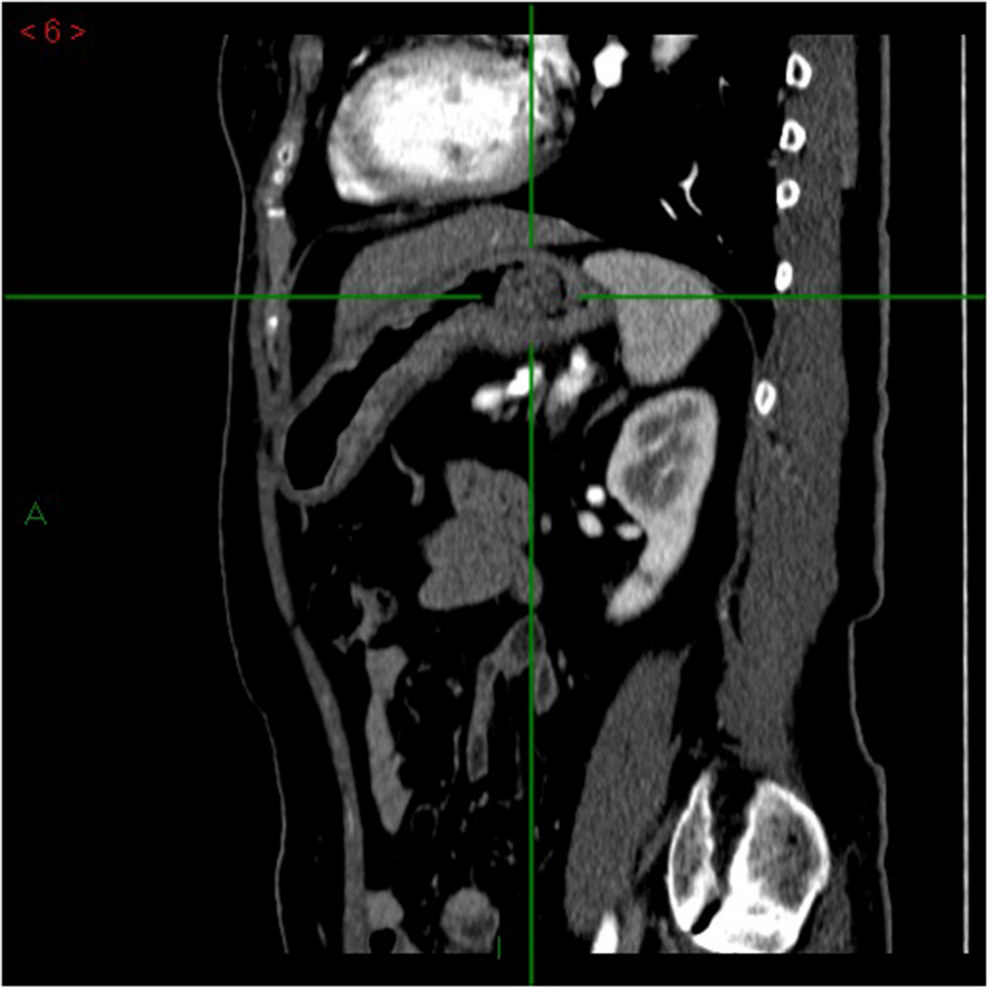
Multidetector computed tomography (MDCT) arterial-phase, sagittal plane. Focal mass in the gastric lumen, next to the cardias, characterized by enhancement comparable to that of the gastric wall. The mass is vegetating into the gastric lumen.

Since August 2021, the patient has been complaining of mild and transitory dyspepsia that did not cause nocturnal awakening. These symptoms were considered as non-troublesome and did not prompt an immediate medical evaluation. However, she progressively lost 6 kg and, in late September, dysphagia for both solid food and liquids occurred, with nausea and a few vomiting episodes. For these reasons, an upper gastrointestinal endoscopy was performed, showing, in stomach retroflection, a polypoid sessile, though irregular, lesion (Paris classification 0-Is) of roughly 3 cm, easily bleeding, with a inhomogeneous mucosal pattern, in the fundus of the stomach, close to the cardia. The lesion was also observed with narrow band imaging, showing a deeply altered mucosa, with an irregular pattern, architectural distortion, and irregular vascularization. Consistently, biopsies from the lesion showed high-grade epithelial dysplasia. At this first evaluation, esophageal biopsies were not taken, as the cause of dysphagia was clearly attributed to the lesion found in the cardia region. Additionally, biopsies from normal-appearing gastric mucosa were not taken due to the bleeding secondary to the biopsy sampling of the lesion. Given the endoscopic suspicion of gastric cancer, a multidetector CT scan was prescribed, showing a 3-cm polypoid mass in the stomach fundus, involving the cardia area (Figure 1). No pathological lymph nodes in the abdomen, around the stomach and esophagus, were noticed, nor were metastases noticed in the abdominal parenchymal organs. An endoscopic ultrasound was also performed, showing a 3-cm solid, inhomogeneous mass, reaching the Z line of the esophagus, with no pathological lymph nodes (T2N0 lesion).

**Figure 2:**
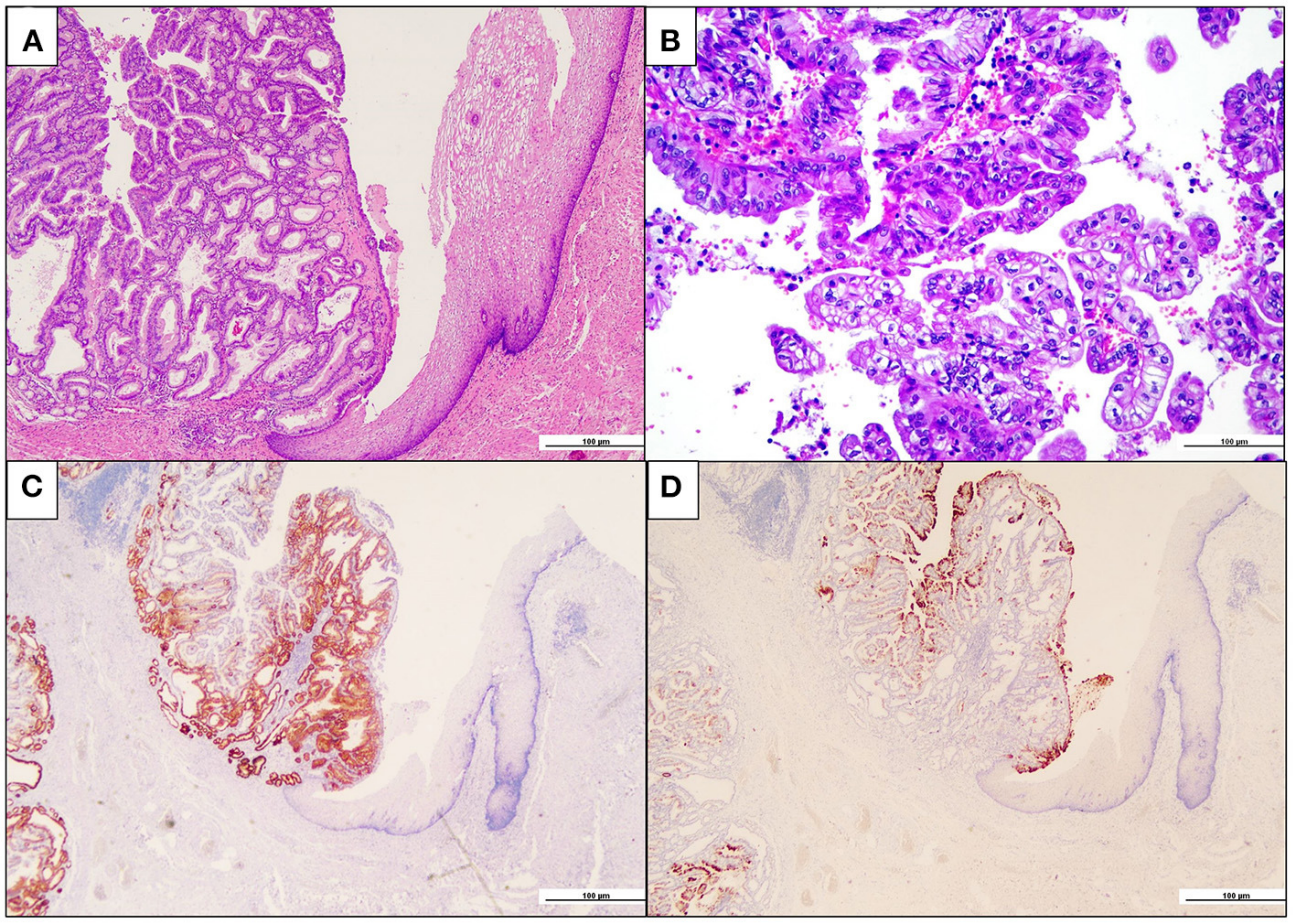
Pyloric gland adenoma of the cardia. **(A)** The intramucosal neoplastic proliferation (on the left) is composed of closely packed glandular units. Note, on the right, the esophageal squamous epithelium (hematoxylin and eosin). **(B)** Focally, high-grade dysplastic features, with marked loss of nuclear polarity and complex papilary architecture, are seen (hematoxylin and eosin). **(C)** Neoplastic glands are diffusely immunoreactive for the pyloric gland marker MUC6 (MUC6 immunohistochemistry). **(D)** A superficial coating of MUC5AC-positive cells is appreciated, while the deeper glands are MUC5AC-negative (MUC5AC immunohistochemistry).

**Figure 3:**
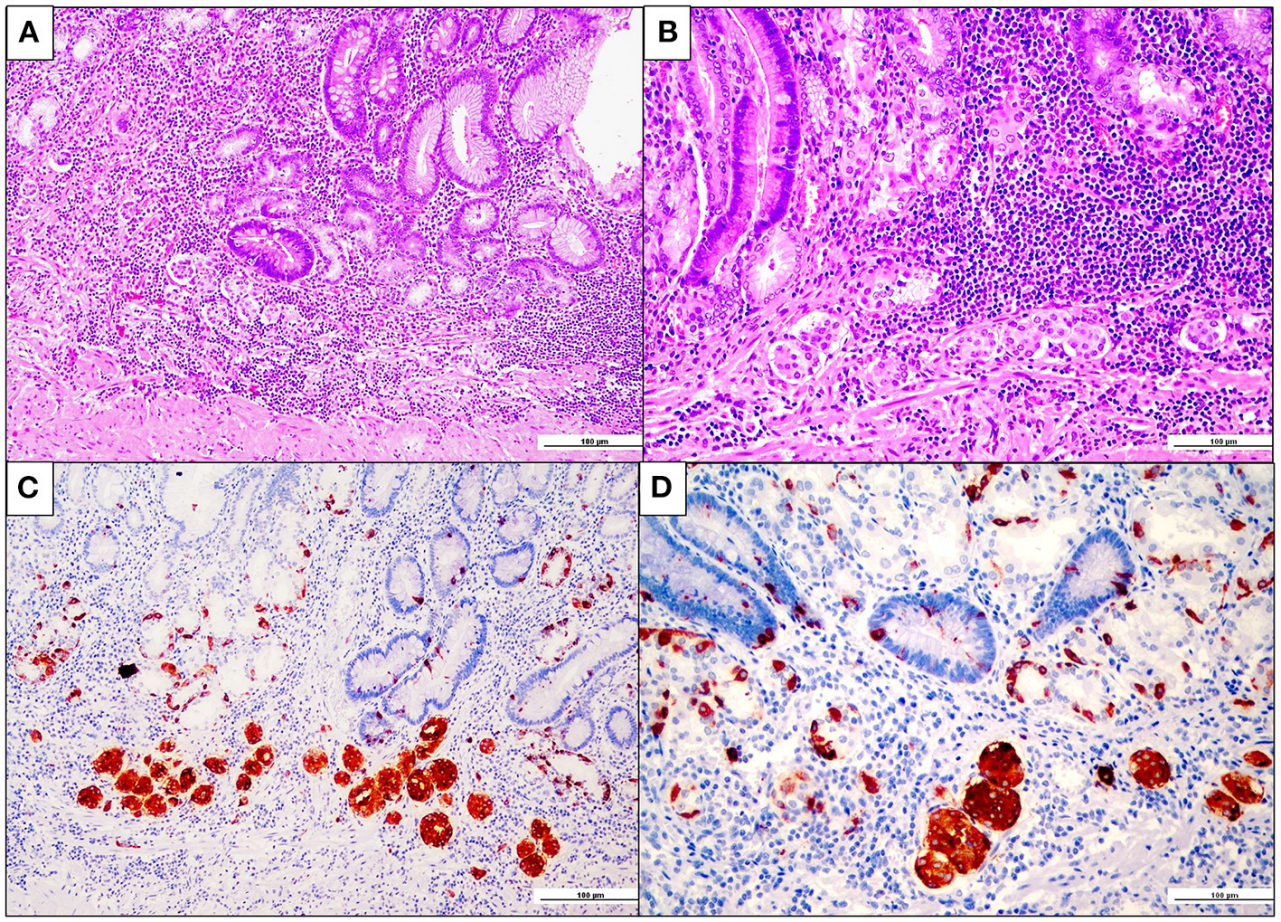
Fundus enterochromaffin-like (ECL) cell hyperplasia and dysplasia. **(A,B)** Oxyntic mucosa showing severe atrophy with extensive pseudopyloric and intestinal metaplasia. Note the presence of aggregates of closely packed micronodules in the deep lamina propria, at higher magnification in B (hematoxylin and eosin). **(C)** Chromogranin-A-positive ECL cell adenomatoid hyperplasia, consisting of aggregates of more than four neuroendocrine cell micronodules in the lamina propria (chromogranin-A immunohistochemistry). **(D)** ECL cell fused micronodules (with no intervening basal membrane), consistent with neuroendocrine cell preneoplastic/dysplastic lesions (chromogranin A immunohistochemistry).

Following the imaging studies, the patient underwent a proximal gastrectomy, considering that an endoscopic approach was contraindicated due to both the localization of the lesion and the staging. Upon gross examination, the gastric mucosa showed a polypoid, mass-forming 3-cm lesion, centered on the cardia, just below the gastro-esophageal junction. The lesion was composed of closely packed pyloric-like glands, mainly lined by columnar cells with round nuclei and inconspicuous nucleoli, and glassy-to-eosinophilic cytoplasms (Figure 2A). Focal areas of increased architectural complexity with loss of cell polarization, consistent with high-grade dysplasia, were also seen (Figure 2B). Immunohistochemistry was performed, and the lesion was diffusely immunoreactive for cytokeratin 7 (monoclonal, clone OV-TL, 12/30, Dako Omins) and negative for cytokeratin 20 (monoclonal, clone Js20.8, Dako Omnis) and CDX2 (monoclonal, clone DAK-CDX2, Dako Omnis). Expression of the mismatch repair proteins was retained. The glandular proliferation expressed MUC5AC (monoclonal, clone CLH2, Dako Omnis) in the superficial component and MUC6 (monoclonal, clone CLH5, Novocastra) (Figures 2C,D) in the deeper portion. The lesion was entirely submitted for histological analysis. Very limited areas suspicious for lamina propria infiltration were observed, whereas no submucosa invasion was found. A final diagnosis of PGA of the gastric cardia was made. Esophageal mucosa was histologically unremarkable. On the contrary, the oxyntic mucosa adjacent to the PGA featured the histological hallmarks of autoimmune atrophic gastritis: severe mucosal atrophy with extensive intestinal and pseudo-pyloric metaplasia, chronic inflammation, absence of Helicobacter pylori, and linear and micronodular hyperplastic proliferations of the ECL cells (Figures 3A,B). In addition, adenomatoid hyperplasia, defined as clusters of 5 or more ECL-cell micronodules in the lower third of the mucosa (Figure 3C), as well as dysplastic ECL-cell proliferations (fused micronodules without intervening basal membrane) were identified (Figure 3D). No neuroendocrine tumor was seen in the resected specimen. After surgery, the patient recovered promptly, and dysphagia completely disappeared. After roughly 1 month, the patient complained of postprandial fullness, which is well controlled by the use of a prokinetic and by dietary habits modifications (i.e., eating slow, small but frequent meals, soft food). Given the suspicion of AIG, an upper gastrointestinal endoscopy with multiple gastric biopsies according to the updated Sydney-Houston criteria (13) was later performed, and the diagnosis of AIG was histologically confirmed (i.e., severe corpus atrophy sparing the antrum, linear and micronodular ECL cell hyperplasia in the corpus mucosa and gastrin cell hyperplasia in the antrum; H. pylori-negative). Finally, the patient was tested for serum anti-parietal cell antibodies, that turned out to be positive (>100 U/mL, ELISA), fasting 17-gastrin (3,122 pg/ml; normal value <98 pg/ml), and chromogranin A (133 ng/ml; normal value <100 ng/ml). Lifelong parenteral vitamin B12 supplementation was recommended (14), and proton pump inhibitors were contraindicated. The clinical course of the patient is summarized in Table 1.

**Table 1:** Timeline of the disease and treatment.

**Timeline**	**Symptoms**	**Diagnosis and treatment**	**Outcome**
August 2021	Mild dyspepsia	None	
Late September 2021	6-kg weight loss, solid and liquid dysphagia, nausea, vomiting	Upper gastrointestinal endoscopy: biopsies from a cardiac 3-cm mass showing high-grade gastric dysplasia. Proximal gastrectomy: cardiac high-grade PGA, in a context of AIG with ECL cell hyperplasia and dysplasia	Improved control of symptoms
December 2021	Post-prandial fullness	Upper gastrointestinal endoscopy confirmed AIG without evidence of neoplasia/dysplasia. Therapy: prokinetic, dietary habit modification, parenteral vitamin B12 supplementation	Improvement of symptoms

*AIG, autoimmune gastritis; PGA, pyloric gland adenoma; ECL, enterochromaffin-like*.

## Discussion

Gastric atrophy, either autoimmune or Helicobacter pylori-induced, may alter the microbiota, by impairing acid secretion, and may result in a potentially genotoxic “atrophy-associated microbiome”, which may promote a microenvironment prone to development of gastric dysplastic/neoplastic lesions (5, 15, 16). In addition, hypergastrinemia resulting from hypochlorhydria may contribute to the onset of neoplastic proliferations. This is particularly relevant for ECL cell neuroendocrine tumors, as long-standing hypergastrinemia is known to cause selective gastrin-dependent ECL cell hyperplasia, which may progress to ECL cell dysplastic and neoplastic proliferations (3, 17). On the contrary, the risk of gastric adenocarcinoma associated with autoimmune gastritis remains poorly known and, although increased compared to the general population (18, 19), it is likely to be low, especially when the etiological involvement of Helicobacter pylori and association with Lynch syndrome are excluded (5, 20, 21). Autoimmune gastritis-related pseudopyloric metaplasia, which by itself (i.e., without concomitant intestinal metaplasia) seems not to be associated with an increased risk of gastric cancer (22), is considered the putative precursor lesion of the rare PGA, which, in turn, may progress to gastric adenocarcinoma (11). Kushima et al. found that PGAs share common genetic and phenotypic features with gastric adenocarcinoma of the fundic gland type, suggesting that these neoplasms are closely related entities with a common mucous neck cell/immature chief cell lineage phenotype (23). This hypothesis is very intriguing as, in murine models, the bulk of spasmolytic polypeptide-expressing metaplasia (SPEM), which is the “molecular” counterpart of pseudopyloric metaplasia, has been shown to arise from chief cells *via* a process called “paligenosis” (i.e., chief cell transdifferentiation) (24, 25). Whether pseudopyloric metaplasia arises from chief cells also in human autoimmune gastritis and may subsequently evolve to PGA deserves further studies.

A peculiar feature of the case here described is the PGA location in gastric cardia, which might appear as an unusual site for PGA development. However, it should be recalled that (i) in AIG, anatomic cardia may be involved by the same pseudopyloric and/or intestinal metaplastic mucosal changes of gastric corpus-fundus, as seen in the present case, and (ii) PGAs, despite their name, exhibit phenotypical and immunophenotypical features more similar to mucous neck cell lineage of oxyntic glands rather than to pyloric gland cells (23). Indeed, Vieth and Montgomery previously reported that 17 and 13% of gastrointestinal tract PGAs from their institutions in Germany (Bayreuth) and the USA (Baltimore), respectively arose in the cardia (12).

Another relevant histologic lesion found in the specimen was the so-called “ECL cell dysplasia”, a poorly known form of neuroendocrine cell proliferation in the oxyntic mucosa, falling short of the diagnosis of gastric NET (2, 26). ECL cell dysplasia has been defined as enlargement and fusion of ECL cell nodules measuring <0.5 mm in diameter and encompasses four histologic patterns, i.e., enlarged micronodules, fused micronodules, micro-infiltrative lesions and nodules with newly-formed stroma (2, 3, 27). It has been described and found to be related with concomitant and/or subsequent detection of type 1 (associated with chronic atrophic gastritis) and type 2 (associated with Zollinger-Ellison syndrome in the context of multiple endocrine neoplasia type 1) ECL cell NETs (3, 28, 29). On the other hand, neither ECL hyperplasia nor ECL cell dysplasia have been described in type 3 (sporadic) gastric NETs (27). Recently, two additional types of gastric neuroendocrine tumors have been described in the oxyntic mucosa, both associated with hypergastrinemia and ECL cell hyperplastic proliferations, similarly to type 1 and type 2 NETs (30). Type 4 gastric NETs seem to be very rare and their pathogenesis seems to be related to a defect of the proton pump function due to an inactivating mutation of ATP4A gene (31, 32), whereas the new, albeit yet provisional, category of type 5 ECL cell NETs has been defined as ECL cell NETs occurring in a background of non-atrophic fundic mucosa of patients receiving long-term treatment with proton pump inhibitors and hypergastrinemia (30, 33). Although ECL cell hyperplasia has been found in both type 4 and type 5 gastric ECL cell NETs, ECL cell dysplastic changes have been reported in type 4 ECL cell NETs only, in addition to type 1 and 2 NETs.

## Conclusion

To conclude, we have herein reported an unusual case of two different-and concurrent-gastric cancers involving an atypical gastric region (i.e., cardia) in a patient with AIG. It is known that AIG is burdened by a substantial diagnostic delay due to its proteiform clinical manifestations (14), especially in case of previous misdiagnosis or rare manifestations due to vitamin B12 deficiency. In this case, a diagnosis of AIG could have possibly been made months or years before, by applying a proactive screening strategy with serology (i.e., serum autoantibodies and fasting 17-gastrin), having the patient an autoimmune thyroid disease which may be associated with AIG in up to 20% of the cases (1). Indeed, a proper upper gastrointestinal endoscopic surveillance is warranted in patients with AIG, with a time interval of 1 to 5 years depending on specific disease and patient characteristics (i.e., family history of gastric cancer, severity and extension of atrophy and metaplasia, personal history of gastric cancer, etc…). Guidelines for the endoscopic follow-up have been recently drafted and should be extensively applied (34).

## Data Availability Statement

The raw data supporting the conclusions of this article will be made available by the authors, without undue reservation.

## Ethics Statement

The studies involving human participants were reviewed and approved by San Matteo Hospital Foundation. The patients/participants provided their written informed consent to participate in this study. Written informed consent was obtained from the individual(s) for the publication of any potentially identifiable images or data included in this article.

## Author Contributions

CG, ML, and CR conceived and designed the case report, contributed to the clinical and pathologic diagnosis, summarized the case, reviewed the literature, and drafted the manuscript. AP contributed to clinical diagnosis and surgical treatment and provided input in the preparation of the manuscript. AG contributed to radiologic diagnosis and provided the radiologic image. GA contributed to pathology diagnosis and immunohistochemistry. AD and AV supervised the conception, analysis, and manuscript drafting. All authors critically revised the paper for important intellectual content, provided approval of the final version and agreed to be accountable for all aspects of the work, ensuring that questions related to the accuracy or integrity of the work are appropriately investigated and resolved.

## Funding

This work was supported by a Grant of the Italian Ministry of Education, University and Research (MIUR) to the Department of Molecular Medicine of the University of Pavia under the initiative Dipartimenti di Eccellenza (2018–2022).

## Conflict of Interest

The authors declare that the research was conducted in the absence of any commercial or financial relationships that could be construed as a potential conflict of interest.

## Publisher's Note

All claims expressed in this article are solely those of the authors and do not necessarily represent those of their affiliated organizations, or those of the publisher, the editors and the reviewers. Any product that may be evaluated in this article, or claim that may be made by its manufacturer, is not guaranteed or endorsed by the publisher.
